# Charging of Vitreous Samples in Cryogenic Electron Microscopy Mitigated by Graphene

**DOI:** 10.1021/acsnano.3c03722

**Published:** 2023-08-02

**Authors:** Yue Zhang, J. Paul van Schayck, Adrián Pedrazo-Tardajos, Nathalie Claes, Willem E. M. Noteborn, Peng-Han Lu, Hans Duimel, Rafal E. Dunin-Borkowski, Sara Bals, Peter J. Peters, Raimond B. G. Ravelli

**Affiliations:** † Maastricht MultiModal Molecular Imaging Institute (M4i), Maastricht University, 6200 MD Maastricht, The Netherlands; ‡ Electron Microscopy for Materials Science (EMAT), University of Antwerp, Antwerp 2020, Belgium; § NANOlab Center of Excellence, University of Antwerp, 2020 Antwerp, Belgium; ∥ Netherlands Centre for Electron Nanoscopy (NeCEN), Leiden University, 2300 RS Leiden, The Netherlands; ⊥ Ernst Ruska-Centre for Microscopy and Spectroscopy with Electrons and Peter Grünberg Institute, Forschungszentrum Jülich, 52425 Jülich, Germany

**Keywords:** charging, vitreous samples, single-particle analysis, graphene, cryogenic transmission electron microscopy

## Abstract

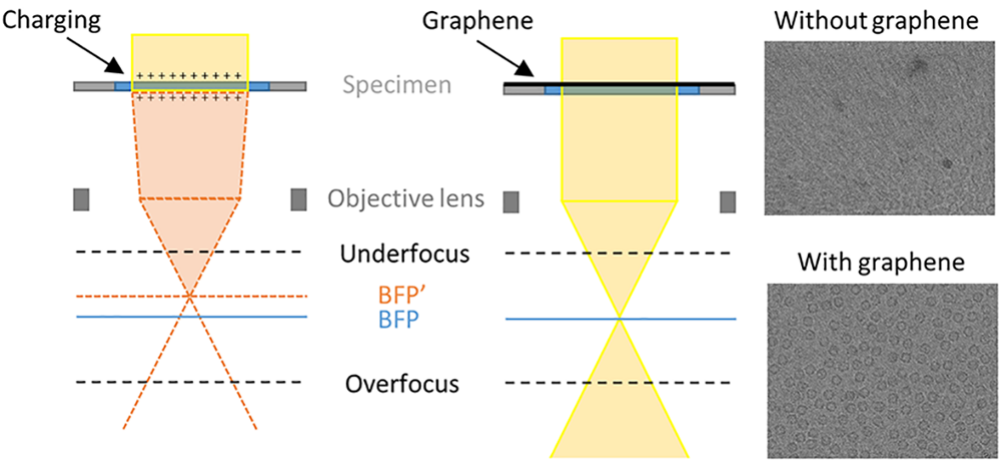

Cryogenic electron microscopy can provide high-resolution reconstructions of macromolecules embedded in a thin layer of ice from which atomic models can be built *de novo*. However, the interaction between the ionizing electron beam and the sample results in beam-induced motion and image distortion, which limit the attainable resolutions. Sample charging is one contributing factor of beam-induced motions and image distortions, which is normally alleviated by including part of the supporting conducting film within the beam-exposed region. However, routine data collection schemes avoid strategies whereby the beam is not in contact with the supporting film, whose rationale is not fully understood. Here we characterize electrostatic charging of vitreous samples, both in imaging and in diffraction mode. We mitigate sample charging by depositing a single layer of conductive graphene on top of regular EM grids. We obtained high-resolution single-particle analysis (SPA) reconstructions at 2 Å when the electron beam only irradiates the middle of the hole on graphene-coated grids, using data collection schemes that previously failed to produce sub 3 Å reconstructions without the graphene layer. We also observe that the SPA data obtained with the graphene-coated grids exhibit a higher *b* factor and reduced particle movement compared to data obtained without the graphene layer. This mitigation of charging could have broad implications for various EM techniques, including SPA and cryotomography, and for the study of radiation damage and the development of future sample carriers. Furthermore, it may facilitate the exploration of more dose-efficient, scanning transmission EM based SPA techniques.

## Introduction

Stimulated by the Resolution Revolution in cryogenic electron microscopy (cryo-EM),^1^ scientists have made spectacular progress in pushing the limits of SPA to resolve the structure of biological macromolecules, in terms of both resolution and particle size. SPA has yielded atomic resolution reconstructions,^2,3^ as well as reconstructions from particles smaller than the 38 kDa theoretical size limit.^4,5^ SPA data collection has become much faster over the last few years with the availability of faster detectors, advanced microscope automation, fringe-free imaging, aberration-free image shift, and hole clustering.^6^ This higher throughput has been combined with schemes to improve signal-to-noise ratios (SNR), such as DQE improvement of detectors, energy filters, a lower energy spread of the electron source, enhanced phase contrast, and reduced sample movements.

Practically, it is well-known that upon irradiation by electrons, biomolecules embedded in a thin hole-spanning vitreous ice layer are observed to move.^7^ However, the physical mechanisms behind the sample movement are not fully understood. Several hypotheses have been proposed to explain this beam-induced motion.^8^ It has been argued that the sample is placed under compressive stress upon rapid cryocooling. Electron radiation can induce creep in the presence of this stress that results in doming of the sample in the foil openings.^9^ Another hypothesis implicates new mechanical stress from the breakage of chemical bonds and the generation of hydrogen gas. Alternatively, electrostatic charging generates an attractive force that causes bending and warping of the thin flexible sample layer. Furthermore, it has been shown that biomolecules can also appear to shift without physically moving themselves.^10^ Nonideal and dynamically changing lens conditions would result in image distortions in which molecules appear to move.^11^ No matter the cause, movement or distortion during imaging leads to a reduced image quality by dampening high-resolution signals. Several schemes have been proposed to reduce beam-induced motions, such as devitrification,^12^ vitrification at a low cooling rate and elevated temperatures,^9,13^ or the use of grids with small holes to have a low ice thickness/hole diameter ratio.^14^

Charging of biological samples within the TEM has been discussed for decades.^10,15−20^ The ionizing electron beam produces secondary electrons that escape from the sample, thus leaving a positive charge on nonconductive specimens.^17,20−24^ Charging can result in unwanted contrast changes, known as the “bee swarm effect”, characterized by fluctuating granularity caused by random surface charging at low magnification and high-defocus conditions.^21,25,26^ Russo and Henderson described that sample charging is a dynamic process that results from the poor conductivity of the specimen under low electron flux conditions.^27^ The positively charged “footprint” from electron irradiation forms a microlens on the sample, which deflects incoming electron beams, causing a change in phase contrast. This effect, known as the “Berriman effect”, fades when the beam scans nearby regions.^22^ The microlens can already be formed within a fluence range of ∼10^–3^ to ∼1 e^–^/Å^2^ and may contribute to the defocus change observed in the early frames of micrographs.^19,20,28^

Sample charging is normally alleviated by including part of the supporting conducting film within the region exposed to the electron beam.^8,10,22^ Curtis and Ferrier noticed that the “bee swarm effect” does not happen when part of the beam hits the metal grid bar even when the carbon film and the grid bar within the field of view are not connected.^21^ Berriman and Rosenthal designed a special seven-hole C2 aperture and demonstrated that secondary electrons emitted from adjacent areas can reduce the charge on the area of interest.^10^ Objective apertures can also reduce specimen charge: secondary electrons emitted by the objective aperture can compensate some of the positive charge of the surface of the specimen.^16^

One successful technique to reduce beam-induced motion is “spot-scan imaging”, which focuses an electron beam to a diameter of ∼1000 Å and scans it over the specimen to capture multiple images.^29−31^ However, this technique would cause charging on a thin hole-spanning vitreous ice layer. This technique was successful only with specimens supported by a continuous conductive carbon film, which is undesirable for SPA due to the SNR reduction.

A suitable alternative to amorphous carbon is graphene, which has orders of magnitude higher conductivity than a carbon layer,^32,33^ gives minimum background noise, and can be overlaid on top of a support layer. Graphene is used in materials science, and it is increasingly applied in the life sciences.^32,34−40^ Ultraflat graphene can result in uniform thin ice layers, allowing for high-resolution structure determination of sub 100 kDa proteins.^38^ Others described that graphene can be functionalized to improve particle density and orientation.^41−43^

In this paper, we investigate charging effects on vitreous biological specimens and demonstrate that charging can be mitigated by depositing a graphene layer on regular EM grids. We present high-resolution, conventional-TEM SPA reconstructions obtained with such grids and discuss the importance of understanding charging for future conventional and nonconventional SPA schemes. Being able to mitigate charging by deploying graphene could help to further push the boundaries of resolving the high-resolution structures of biomolecules via EM.

## Results

### Evaluating the Effect of Charging in Defocused Diffraction Mode

**Figure 1:**
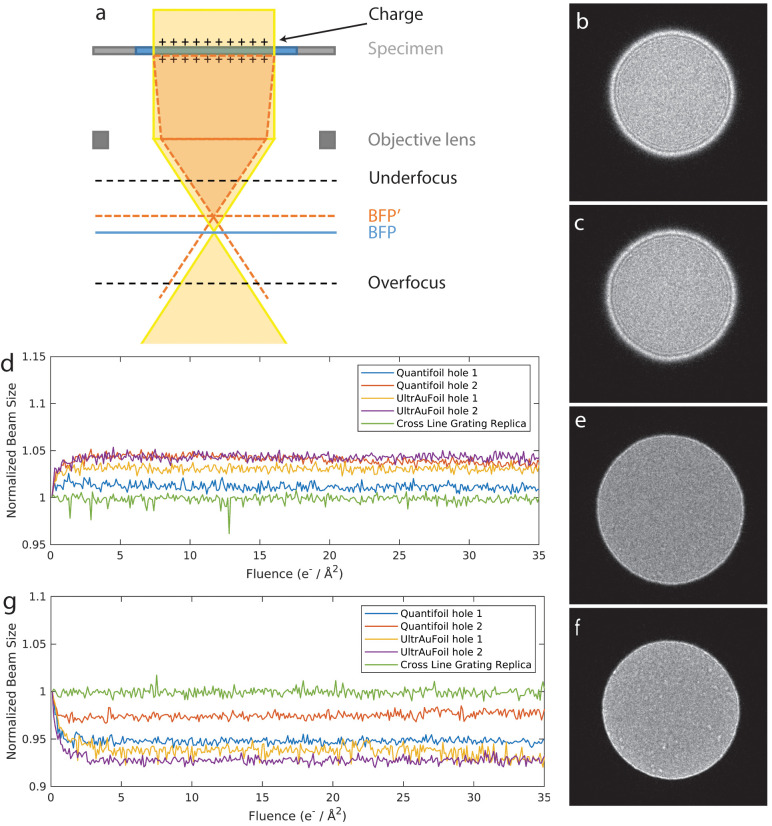
Effect of specimen charging on both Quantifoil and UltrAuFoil shown by DIFF images. (a) Ray diagram of the electron-optical effect of charge on the specimen. The electron beam (solid lines), which irradiates a specimen, is focused by the objective lens at the back focal plane (BFP). Charge on the sample acts as a lens that converges the beam (dash lines) and induces the expansion of the overfocus pattern (b, c) and the shrinkage of the underfocus pattern (e, f). DIFF images before (b, e) and after (c, f) 2 e^–^/Å^2^ irradiation of the specimen. The normalized beam radius is plotted as a function of accumulated dose under overfocus (d) and underfocus (g) conditions for both Quantifoil (blue, red curves) and UltrAuFoil (yellow, purple curves). For comparison, the normalized beam radius as a function of accumulated dose from cross line grating replica (Au) samples is shown (green curve). The electron beam flux on the sample was 0.38 e^–^/(Å^2^ s).

We used regular SPA samples and grids: *Mycobacterium tuberculosis* ferritin (BfrB)^44^ applied to glow-discharged R1.2/1.3 Quantifoil and UltrAuFoil grids. The vitreous ice within the holes of this perforated film acts as an insulator, and a beam size smaller than those of these holes was used. Similar to Brink et al.,^22^ we used a defocused diffraction image (DIFF image) to observe the effect of charging (Figure 1a). A hybrid pixel Timepix3 detector^45−47^ was used to record such images. No objective aperture was used in the DIFF image/movie collection. As a function of fluence, the size of the defocused diffracted beam increased under the overfocused condition (Figure 1b,c and Supplementary Movies 1 and 2) and decreased in the underfocused condition (Figure 1e,f and Supplementary Movies 3 and 4). The normalized DIFF beam size in both overfocus (Figure 1d) and underfocus (Figure 1g) became stable at a fluence of around 1.5 e^–^/Å^2^. The change of the DIFF beam size seems to be insensitive to the type of foil material (carbon or Au), given that the beam is inside the hole. By comparison, the normalized DIFF beam sizes from a conductive crossline grating replica sample (Supplementary Movies 5 and 6) remained constant throughout irradiation.

**Figure 2:**
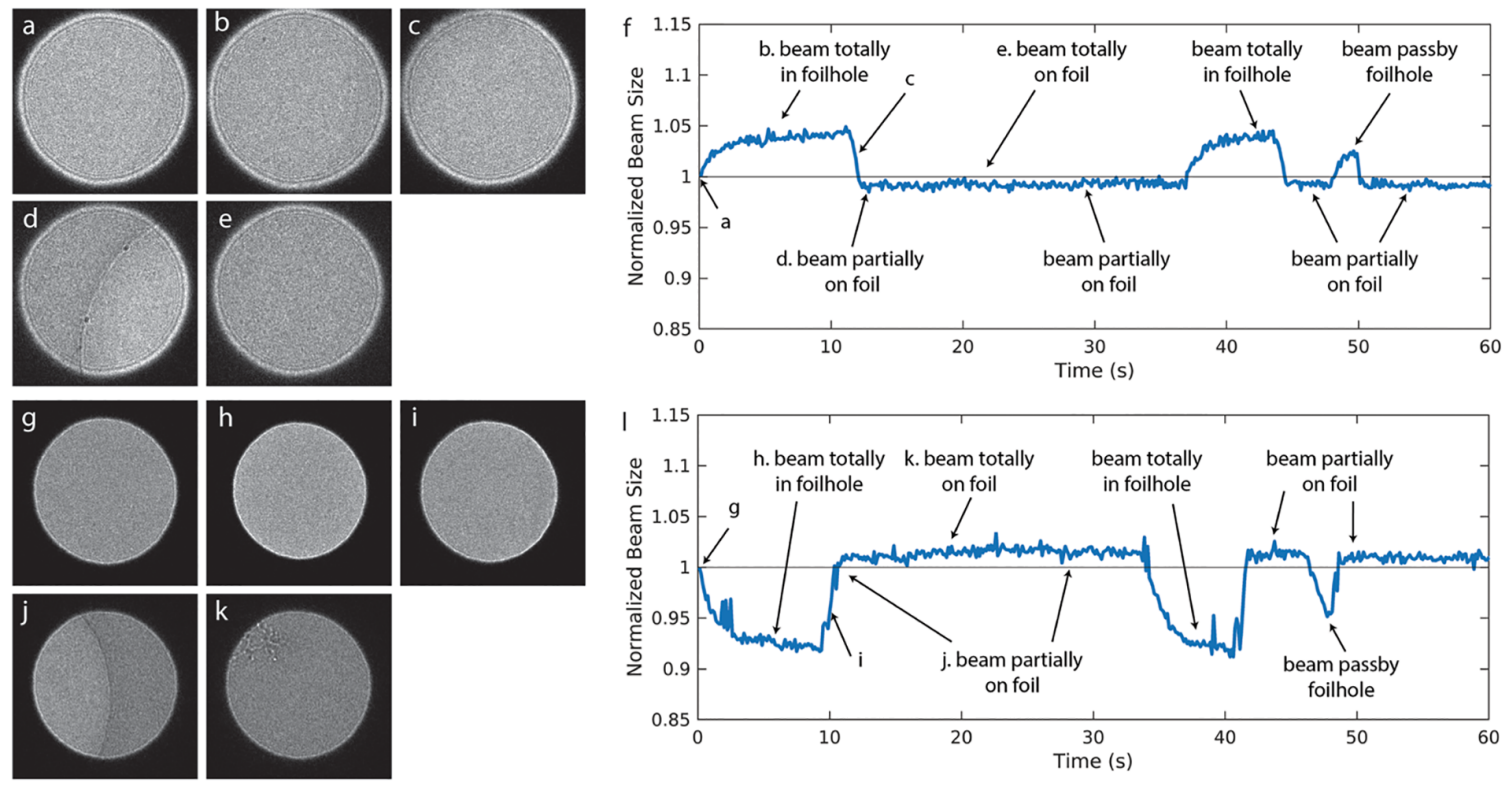
Change of DIFF image beam size upon stage move on Quantifoil grids under overfocus (a–f) and underfocus (g–l) conditions. (f, l) The normalized beam radius is plotted as a function of time, with letters corresponding to the panel images shown to the left. Under overfocus condition (a–f), the beam was completely within the foil hole on ice before irradiation (a), increased after a few seconds of irradiation (b), and then decreased when the carbon foil moved close to but not on the beam edge (c). The size of the DIFF image decreased to its minimum when the beam partially hit the carbon foil (d) and stayed the same when the beam was completely on the carbon foil (e). (g, h) Under the underfocus condition, a similar but inverse trend was observed. The DIFF image size decreased a few seconds after irradiation (h) and increased when the carbon foil moved close to the beam edge (i, j), finally reaching its maximum when the beam hit the carbon foil (j, k). The electron beam flux on the sample was 0.38 e^–^/(Å^2^ s).

To provide a more in-depth observation of how samples are charged and discharged, we collected DIFF movies with a moving stage staying at each location for 5 s (fluence at 1.9 e^–^/Å^2^). At the beginning of irradiation, the diffraction lens was set in overfocus condition, and the beam was confined within a hole on the vitreous sample (Figure 2a) until the DIFF image beam size fully expanded and became stable (Figure 2b). After that, the stage was moved so that the carbon foil came close to the beam. The DIFF image beam size started to decrease when the beam and foil were in close proximity (100–150 nm), yet not overlapping (Figure 2c). The DIFF image beam size decreased to its minimum when the beam was partially on the supporting foil (Figure 2d) and remained at this minimum even when the beam was completely on the foil (Figure 2e). The normalized DIFF beam size as a function of time (Figure 2f and Supplementary Movie 7) highlights the variation in beam size at various beam locations. There is a noticeable bump at around 50 s, reflecting a rapid charge–discharge process as the beam is scanned from one side of the foil to the other. DIFF movies collected in the same manner at a diffraction lens under underfocus condition (Figure 2g–l and Supplementary Movie 8) showed a similar yet inverse trend, wherein the DIFF image beam reached its maximum (instead of minimum) and remained stable near/on the foil and then decreased (instead of increased) in holes. We repeated these experiments with UltrAuFoils and found the same trends (Supplementary Movies 9 and 10).

### The Use of Graphene-Coated Grids

**Figure 3:**
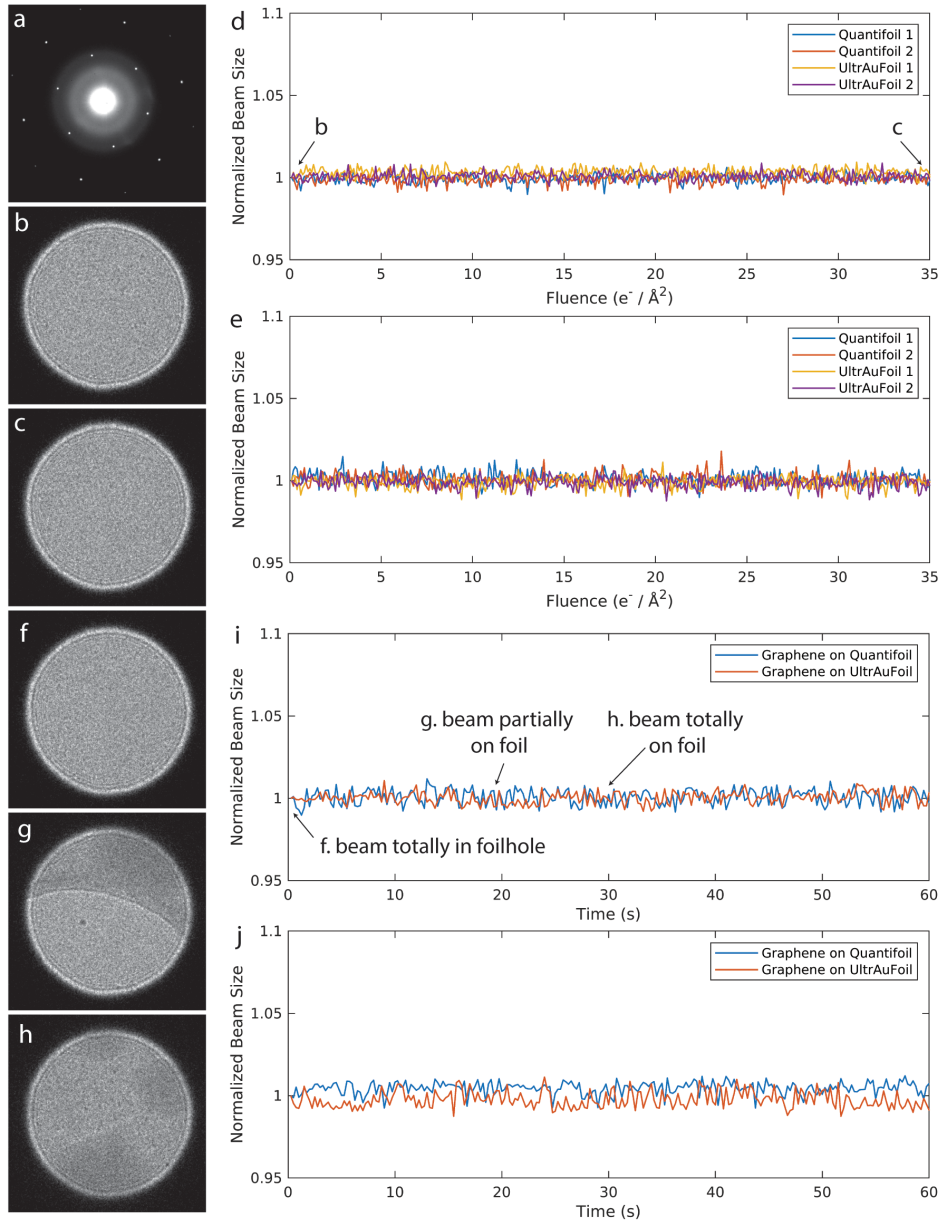
DIFF images of vitreous specimen on Quantifoil with a graphene layer, with similar results being obtained for UltrAuFoil with a graphene layer as well. (a) Diffraction pattern of graphene with vitreous ice on it. (b, c) DIFF images under overfocus conditions are similar before (b) and after (c) 35 e^–^/Å^2^ irradiation. (d, e) The normalized beam radius is plotted as a function of accumulated dose under both overfocus (d) and underfocus (e) conditions for graphene on both Quantifoil (blue, red curves) and UltrAuFoil (yellow, purple curves). When the stage moves, the beam moves from (f) foil hole to (g) partially on the carbon foil to (h) completely on the carbon foil. The DIFF image size was stable as the function of time under (i) overfocus and (j) underfocus conditions for graphene on both Quantifoil (blue curve) and UltrAuFoil (red curve). The electron beam flux on the sample was 0.38 e^–^/(Å^2^ s).

Next, we repeated the experiments above with grids (both Quantifoil and UltrAuFoil) with an extra graphene layer applied on top of them to test whether the conductivity from graphene could alleviate charging. We verified the presence of graphene by collecting electron diffraction patterns from samples with amorphous ice on graphene (Figure 3a). These show the hexagonal diffraction pattern of graphene, demonstrating that it withstood the 10 s glow discharge, sample application, vitrification, and grid handling. The DIFF beam size remained unchanged from the beginning of irradiation (Figure 3b) up to a fluence of 35 e^–^/Å^2^ (Figure 3c), under both overfocus (Figure 3d) and underfocus (Figure 3e) conditions. The DIFF movies with graphene grids are shown as Supplementary Movies 11–14. The DIFF beam size also remained stable for the DIFF image with a moving stage. The normalized DIFF beam size (Supplementary Movies 15–18) as a function of time under both overfocus (Figure 3i) and underfocus (Figure 3j) conditions showed that it remained unchanged regardless of beam location, whether inside the foil hole (Figure 3f), partially on carbon foil (Figure 3g), or totally on carbon foil (Figure 3h). Notably, the location of the graphene layer (on top of/underneath the vitreous ice in the microscope) did not affect the results (data not shown).

### Charging in Imaging Mode

**Figure 4:**
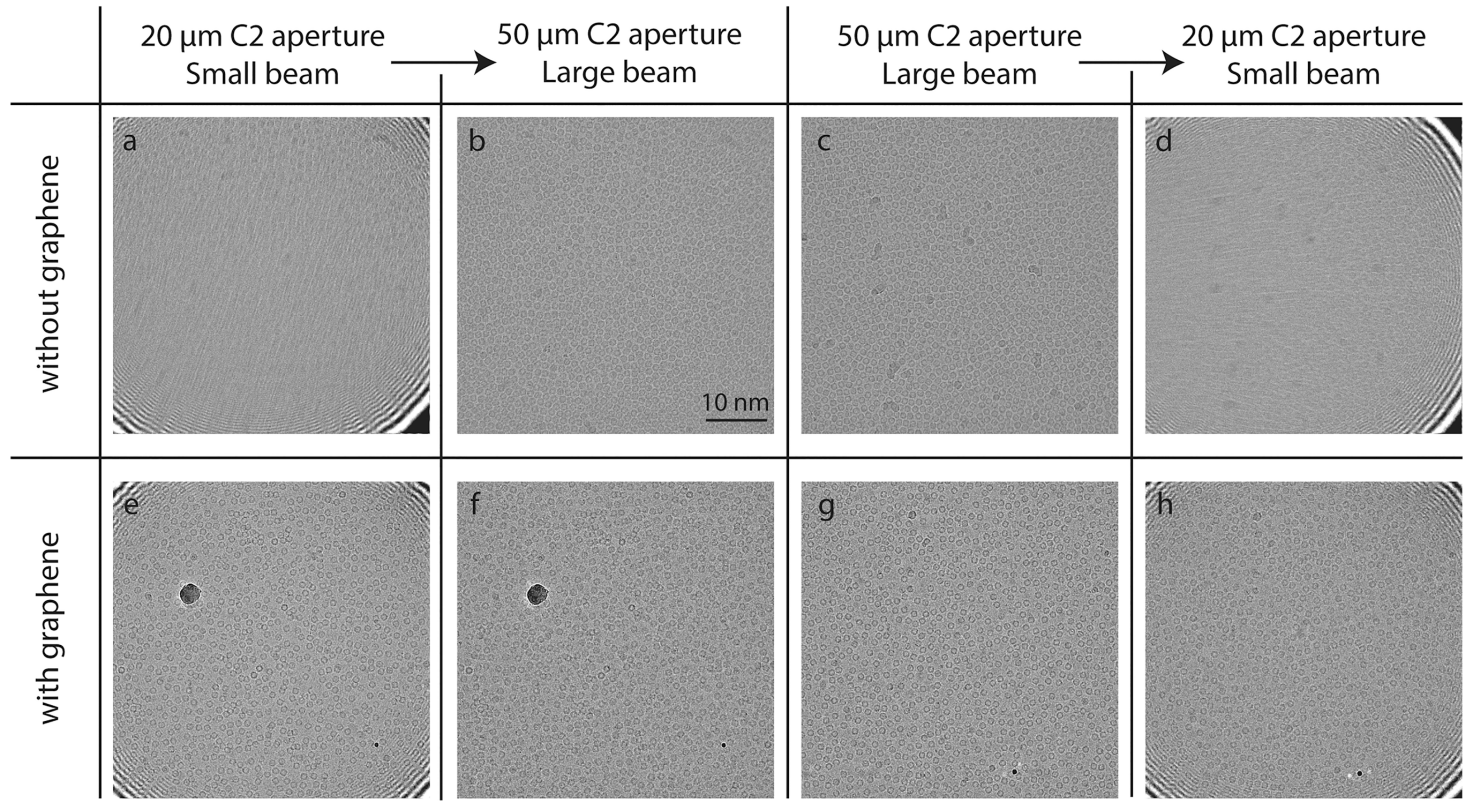
Micrographs of a BfrB sample collected at a magnification of 78000× on a Falcon III detector at 200 kV. All micrographs were collected at a flux of 30 e^–^/(Å^2^ s) for 1 s, and fractions were averaged without motion correction. No objective aperture was used. Micrographs (a–d) have samples at the concentration of 50 mg/mL on normal Quantifoil grids, and micrographs (e–h) show samples at the concentration of 5 mg/mL on Quantifoil grids with a graphene layer. The grids have foilhole size of 1.2 μm in diameter. (a, b) Two successive micrographs at the same position on Quantifoil grid, collected with a 20 μm C2 aperture, beam size of ∼800 nm for 1 s irradiation (a), and then with a 50 μm C2 aperture, beam size of ∼1.9 μm, for 1 s irradiation (b). (c, d) Two successive micrograph pairs at the same position on Quantifoil grids but first collected with a 50 μm C2 aperture for 1 s irradiation, then with a 20 μm C2 aperture for 1 s irradiation. (e, f) Two successive micrographs at the same position on a Quantifoil grid with a graphene layer, first collected with a 20 μm C2 aperture, beam size of ∼800 nm for 1 s irradiation, then with a 50 μm C2 aperture, beam size of ∼1.9 μm, for 1 s irradiation. (g, h) Two successive micrographs at the same position on Quantifoil grids with a graphene layer, but first with a 50 μm C2 aperture for 1 s, then with a 20 μm C2 aperture for 1 s irradiation.

While the DIFF experiments showed clear charging effects up to a fluence of 1.5 e^–^/Å^2^, after which the beam size remained constant, typical SPA data collection schemes conducted in imaging mode use fluences of tens of e^–^/Å^2^, which may be more prone to aberrations that are not readily apparent in diffraction mode. Thus, we used the imaging mode with parallel illumination (Figure 1a) to further investigate charging in these regimes. Images were recorded at the flux of 30 e^–^/(Å^2^ s) for 1 s with a Falcon III detector and no objective aperture at a nominal magnification of 78000× and averaged without patch-track motion correction. We first used a 20 μm C2 aperture to ensure a beam diameter (800 nm) smaller than the hole size (1.2 μm): the beam did not touch the perforated support film. This setup resulted in severely distorted images, as if the particles were moving outward from a center (Figure 4a). After 10 s, another image was taken at the same spot with a 50 μm C2 aperture (beam size 1.9 μm) so that the beam hit the foil. The resulting image was sharp and similar to the initial state (Figure 4b), arguing that the sample had not undergone a plastic deformation but a reversible process only that affected the image. This image distortion (blurring) and restoration (deblurring) are clearly shown in Supplementary Movie 19. To confirm that this effect is not related to the pre-exposure, we collected movies with apertures in the reverse order, first with a 50 μm C2 aperture (Figure 4c) and then with a 20 μm C2 aperture (Figure 4d). We found similar results: a sharp image with the larger aperture and a distorted image with the smaller aperture (Supplementary Movie 20). Importantly, when we performed the same experiments using Quantifoil grids with a graphene layer we consistently obtained sharp images, independent of the size of the beam relative to the foil hole size (Figure 4e,f and Supplementary Movie 21; Figure 4g,h, Supplementary Movie 22).

### Single-Particle Analysis

**Figure 5:**
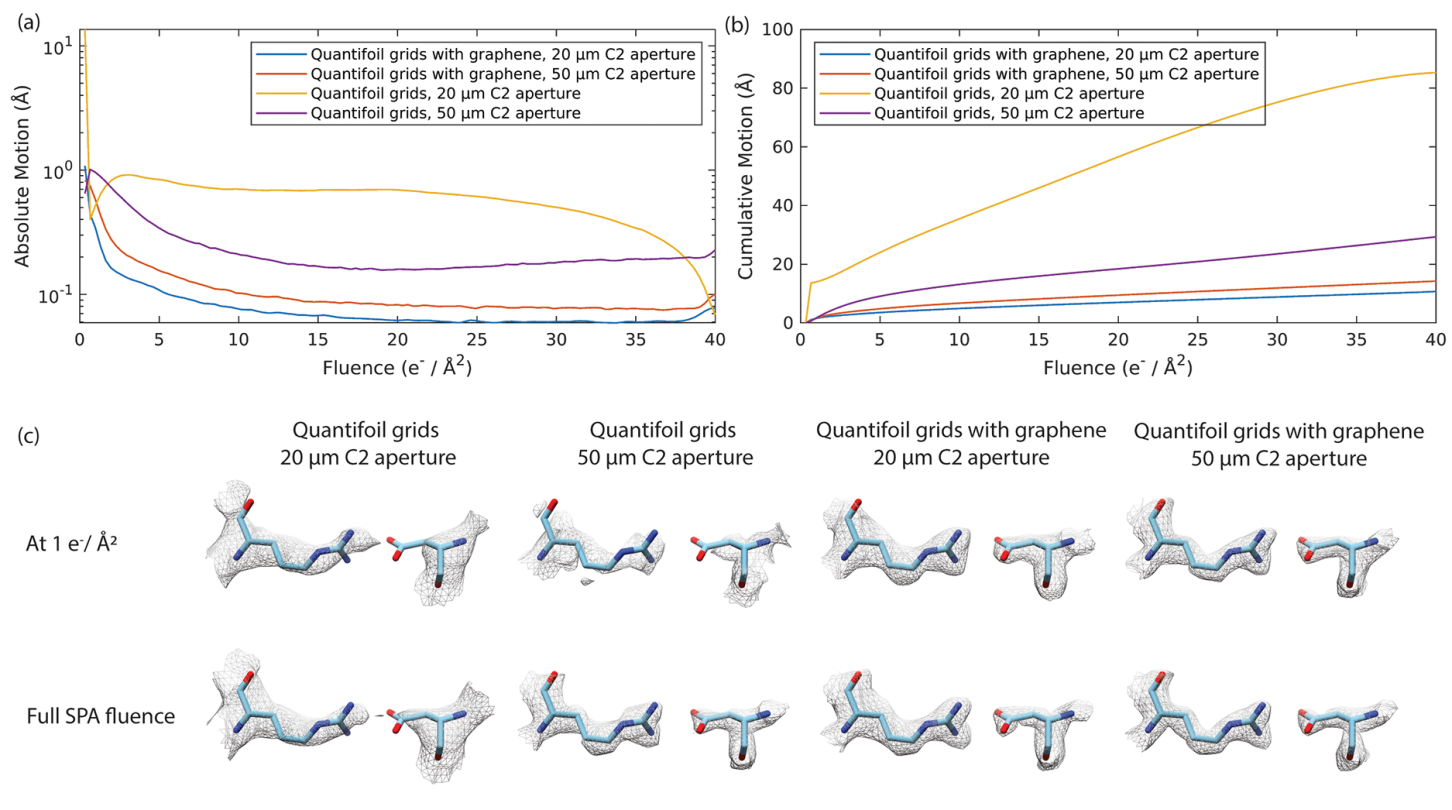
Averaged absolute per-frame motion (a) and averaged accumulated motion (b) as a function of fluence determined by Relion Bayesian polishing of four data sets listed in Table 1. (c) Density maps reconstructed from four data sets, each with a fitted BfrB model (PDB: 7O6E). Reconstructions from 1 e^–^/Å^2^ (top row) and the full SPA fluence up to 40 e^–^/Å^2^ (bottom row). Density maps are drawn at 1.5 RMSD.

Next, we determined whether the use of graphene improved the image quality for SPA structure determination. We collected SPA data sets of BfrB samples on Quantifoil grids with and without graphene (Table 1). Samples on graphene grids exhibited substantially reduced absolute and collective motion compared to samples on grids without graphene throughout the entire SPA fluence period (Figure 5a,b). We observed overlapping particles when using the graphene-coated grids and could use a 10-fold dilution of the protein sample to arrive at a similar number of particles per micrograph when compared to the grids without graphene. Under conditions where the beam is smaller than the hole and the supporting foil is not exposed, we were unable to get a sub 3 Å reconstruction of BfrB using grids without graphene (Supplementary Figure 1). However, we could obtain a 2.01 Å reconstruction with graphene grids (Supplementary Figure 2). When the beam size was larger than the hole size and touched the conductive support, we achieved reconstruction of maps at 2.12 Å resolution with Quantifoil grids without graphene (Supplementary Figure 3) and 1.90 Å resolution with graphene-coated grids using a similar number of particles (Supplementary Figure 4 and Table 1).

**Table 1:** Data Set Statistics

	data set			
	1	2	3	4
grid type	Quantifoil 300 mesh R1.2/1.3	Quantifoil 300 mesh R1.2/1.3 with graphene	Quantifoil 300 mesh R1.2/1.3	Quantifoil *300 mesh R*1.2/1.3 with graphene
microscope	Krios			
voltage (kV)	300			
objective aperture (μm)	100			
nominal magnification (1000×)	105			
physical pixel size (Å)	0.834			
camera	K3			
detector mode	counting			
focus range (μm)	–0.8 to −2.0		–0.6 to −1.6	
exposure time (s)	1.7			
flux (e^–^/(Å^2^ s))	23.5			
fluence (e^–^/Å^2^)	40			
fractions (no.)	122			
beam size (μm)	0.9		1.8	
micrographs (no.)	633	621	2226	1808
protein particles (no.)	85707	146626	596238	494154
symmetry imposed	O			
FSC threshold	0.143			
map resolution (Å)	3.50	2.01	2.12	1.90

## Discussion

In this study, we examined the effect of charging on cryo-EM SPA samples and demonstrated that the addition of a graphene layer could mitigate this effect, resulting in higher resolution reconstructions and allowing for improved SPA data collection schemes.

### Charging of Sample Forms a Nonideal Microlens

We set out to observe the effect of charging on the cryo-EM SPA sample grids. From Brink et al.,^22^ it is known that the effect of charging is particularly noticeable in defocus diffraction mode (DIFF), where it can be observed as a change in the size of the beam (Figure 1a). We found that beam size in DIFF mode became stable after a fluence of ∼1.5 e^–^/Å^2^ (Figure 1d,g), indicating that the charge saturates at this fluence, in accordance with Schreiber’s findings.^20^ The beam size change appears to be insignificantly affected by the foil material used, here carbon and gold. However, the results shown in Figure 2 indicate that they do relate to the distance between the beam edge and foil. In the experiment of Figure 2, the sample stage is moved while recording and the beam size started to change when the foil moved close to the beam edge (100–150 nm), but before the beam hit the foil (Figure 2c,i). We speculate that the sample starts to discharge when the beam edge and foil are in close proximity. The electron irradiation induces a positive charge on the nonconductive sample surface with its area broader than the beam size. This charge produces a three-dimensional potential distribution that extends further in all directions,^17,20^ with an electric field strength of more than a few MV/m.^22,24^ This potential distribution can deflect incoming electrons and cause a drift of the beam (Supplementary Movies 7–10). These observations confirm the conclusions in ref (8), where it was already indicated that charging leads to a deflection of the incident beam and results in the creation of undesirable lenses. Then the sample discharges when the beam hits the foil (Figure 2d,j). Close inspection of the normalized beam size indicates that a nonconducting SPA sample can become charged at an extremely low initial fluence: at the very first frame recorded with 0.047 e^–^/Å^2^ (Figure 2f,l). Overall, the results shown in Figures 1 and 2 demonstrate that the positive charge induced on the surface of the sample by the electron beam forms a nonideal microlens that causes the beam size to change in defocused diffraction mode.

### Charging of the Sample Can Severely Hinder SPA

Surprisingly, whereas the beam size change observed in DIFF mode saturated around 1.5 e^–^/Å^2^, we reproducibly observed continuing distortions in imaging mode for the full range of fluences normally used in SPA (Figure 4a). While distortions were observed in the DIFF image as well—features (e.g., ice contamination) were moving outward even though the DIFF image beam size became stable after 1.5 e^–^/Å^2^ (Supplementary Movie 3)—these distortions were reversible and restored as soon as the beam touched the conductive support film (Figure 4b and Supplementary Movie 19), which we attribute to sample discharging. Performing control experiments in reverse order (Figure 4c,d) showed that the image distortion was not due to pre-exposure. We speculate that, as sample charging is a dynamic process,^27^ the continuing distortion of the image in imaging mode might be attributed to aberration effects, which occur due to charge redistribution on the sample surface, that further distort the image even when the absolute charge of the sample already reached a maximum. Such distortions have hindered routine SPA data collections at the center of the hole using beam sizes smaller than the hole size. The SPA data set we collected in this way displayed severe image distortions (Supplementary Figure 1a), and selecting particles was challenging (Supplementary Figure 1a). Efforts to obtain a proper initial map and reconstruction from this data set were unsuccessful. Despite this, we could eventually obtain a correct BfrB reconstruction utilizing modern motion-correction techniques combined with Bayesian polishing, albeit only at 3.5 Å and a low *b* factor (Supplementary Figure 5). This result not only highlights the power of modern image data processing tools but also provides a warning, as the success of these programs might blind the user to the underlying physical phenomena that prevented them from getting better data.

### Graphene Mitigates Effects of Charging

Both the DIFF image and imaging experiments illustrate that the conductive graphene layer can alleviate charging. The DIFF image beam size was unchanged from the beginning of the irradiation to the full dose typically used in SPA (Figure 3d,e). The averaged images presented no blurring independent of the beam location, whether a small beam illuminated the middle of a hole (Figure 4e) or touched the foil (Figure 4g). With a graphene layer, we were able to obtain a good SPA reconstruction of BfrB at 2.01 Å when the beam hit the middle of the hole (Supplementary Figure 2b). Additionally, for the BfrB protein that we used to perform the experiment, the graphene helped to concentrate the sample within the holes,^42,43^ as we could use a 10 times diluted sample compared to the grids without graphene and still obtained a similar number of particles per micrograph (Table 1). The *b* factor remained relatively unchanged regardless of the beam size for the sample on graphene-coated grids (−82 and −84 Å^2^); however, it was significantly higher compared to that of Quantifoil grids (−178 Å^2^) (Supplementary Figure 5). Despite the *b* factor of the data set without graphene on Quantifoil grids with a large beam (−83 Å^2^) being comparable to data sets with graphene, the resolution values on the *y* axis at the same *x* axis values (number of particles) were lower than the other two data sets, and the standard deviation was high (Supplementary Figure 5). While our results demonstrate that graphene mitigated charging and indeed resulted in improvements in movement and resolution (Figure 5a,b and Table 1), we cannot attribute this improvement to a specific mechanism, whether by suppression of the doming or suppression of the microlens effect. However, it cannot be excluded that graphene may also reduce motion by improving the sample stiffness. To address this question, future experiments involving data collection with tilted samples on graphene-coated grids could provide valuable insight, as it would allow us to observe the movement along the tilt axis and analyze its impact.

When graphene-coated grids were used, particle motions were smaller when a small beam irradiated only the middle of the hole compared to when a large beam irradiated both the vitrified ice and the foil, which might relate to the fact that smaller beams (with same flux) deposit less energy in the sample. The findings are consistent with the “spot-scan imaging” approach described by Downing,^31^ where a small beam was utilized to minimize the beam-induced motion. This technique expands the options for reducing motion beyond solely using small hole grids.^14^

Nevertheless, early-stage, rapid sample motion^8,48^ was observed in Quantifoil grids with and without graphene (Figure 5a), indicating that graphene does not prevent stress release at early exposure. To our surprise, reconstructions of maps obtained from the first fractions of the data were very similar to the final maps for the graphene-coated grids, whereas these early exposure reconstructions show a compromised quality compared with the full exposure reconstructions for grids without graphene (Figure 5c). Several methods have been suggested to improve on the quality of the maps that can be obtained from the initial frames, including devitrification^12^ and vitrification at low cooling rates and elevated temperatures.^13^ However, these techniques may result in the formation of crystalline ice. Although the early-stage motion remains high, our data suggest that the use of graphene can still improve the quality of the data obtained from these first frames. However, further investigation is necessary to fully characterize this effect.

## Conclusions

This study focused on investigating the charging effects on cryo-EM SPA samples and showed that the incorporation of a single graphene layer could effectively alleviate the charging phenomenon with minimal noise. Whether a thicker graphene coating would yield different results will be interesting to explore in future studies. Fabrication of grids with high-quality graphene has been cumbersome due to poor reproducibility, low coverage rate, and contamination.^49^ We, and others,^38^ were able to overcome these limitations by using a high-quality graphene obtained by an improved transfer method where the reproducibility and coverage rate of graphene grids are increased while minimizing contamination.^50^ As a result, these clean graphene grids were able to provide the conductive layer needed to alleviate charging and improve the current SPA schemes. While charging is a fundamental phenomenon in EM, leading to reduced image contrast and resolution for SPA samples, it also applies for tomography lamellae and nonbiological, nonconductive samples. A conductive layer is essential to minimize charging while imaging nonconductive samples, such as biomolecules in vitreous ice, in particular when the beam is not in contact with the conductive supporting layer. A graphene layer provides the good conductivity needed to alleviate charging with minimum background noise. Graphene could also help to reduce radiation damage and provide pristine structures for both materials science and life science samples.^51,52^ Further investigations could be conducted to study the effect of graphene-coated grids on radiation damage in SPA samples, in order to gain a deeper understanding of the electron-induced radiation damage and obtain high-quality structural information on biological molecules. Graphene-coated grids with larger holes could be a promising avenue for future research, allowing for the collection of multiple micrographs with a smaller beam to reduce the beam-induced motion and increase data collection throughput. The use of graphene-coated grids could also potentially improve resolution for other cryo-EM applications, for instance, in the detailed structural analysis of lithium batteries, where better insight into materials, interfaces, and degradation mechanisms^53−55^ could improve the design and optimization of advanced lithium battery systems.

In summary, the implementation of techniques to mitigate charging is a crucial step in improving current SPA schemes as well as providing pristine atomic structures of nonbiological insulating samples. Charging mitigation could enable low-dose imaging or scanning transmission EM (STEM) techniques such as ptychography and iDPC.^56,57^ It is worth noting that the effect of charging may differ in TEM and STEM modes, with local charging and discharging occurring in STEM mode depending on the beam’s position relative to the sample.^58,59^ Further research is necessary to explore the impact of charging in the STEM mode.

## Methods

### Production of Graphene-Coated Grids

Graphene grids were prepared from a commercially available graphene-Cu foil, produced by chemical vapor deposition (CVD). First, a thin layer of cellulose acetate butyrate (CAB) was applied via spin coating in a CAB-ethyl acetate solution. The CAB-graphene-Cu stack was placed on top of an ammonium persulfate etching solution, etching away the Cu. The etching solvent was gradually diluted using deionized water to neutralize the solution and remove the Cu residues from the etched foil. Filter paper was placed at the bottom of the Petri dish, and the grids were placed on top using tweezers. The graphene was transferred onto the grids by removing the water using a micropipet. The filter paper containing the graphene-covered grids was placed on a heating plate for 30 min at 35 °C to dry. Finally, the graphene grids were heated in activated carbon for 15 h at 300 °C, which removed the CAB layer (whose melting temperature is between 170 and 240 °C).

### Sample Preparation for Cryo-EM

*M. tuberculosis* BfrB was prepared according to Gijsbers et al.^44^ and used at a concentration of 50 mg/mL (as calculated with the Pierce BCA Protein Assay Kit) for Quantifoil and UltrAuFoil (Quantifoil Micro Tools, Germany) experiments. We used 300 mesh grids with 1.2 μm diameter holes. A volume of 2.5 mL was applied onto grids that were glow-discharged under vacuum at a current of 7 mA for 30 s. For grids with a graphene layer, a volume of 2.5 μL of diluted BfrB (1:10, 5 mg/mL) was applied onto mildly glow-discharged grids (current of 7 mA for 10 s). Excess liquid was removed by blotting for 3 s using filter paper followed by plunge freezing in liquid ethane using an FEI Vitrobot Mark IV instrument operated under 95% humidity at 4 °C.

### DIFF Image Collection

Diffraction images were collected with a Timepix3 hybrid pixel detector^45−47^ on a 200 keV Tecnai Arctica instrument (Thermo Fisher Scientific). The beam was blocked with the prespecimen beam shutter before exposing the sample for recording. The beam was set to be parallel at a flux of 0.38 e^–^/(Å^2^ s) passing through amorphous ice films. The DIFF image was obtained by defocusing the diffraction lens. No objective aperture was used for the DIFF image data collection. Supplementary Movies are available at: https://doi.org/10.6084/m9.figshare.23244299.v1

### Single-Particle Data Acquisition and Image Processing

Cryo-EM single-particle data were collected on a Titan Krios instrument at 300 kV with a BioQuantum K3 Imaging Filter with a 20 eV postcolumn energy filter. The detector was used in normal counting mode at a nominal magnification of 105000×. Table 1 shows the statistics of the data set. Data were processed using the RELION pipeline.^60^ Movie stacks were corrected for drift (7 × 7 patches) and dose-weighted using MotionCor2.^61^ The local contrast transfer function (CTF) parameters were determined for the drift-corrected micrographs using Gctf.^62^ A first set of 2D references were generated from manually picked particles in RELION,^60^ and these were then used for subsequent automatic particle picking. Table 1 lists the number of particles in the final data set after particle picking, 2D classification, and 3D classification with O symmetry. Beam-tilt parameters, anisotropic magnification, and local CTF parameters were refined, and the particles were polished.^63^ The resolution of the best final map was 2 Å using the gold-standard FSC = 0.143 criterion.^64^ The maps have been deposited in the Electron Microscopy Data Bank as entry EMD-18010, EMD-18028, EMD-18029, EMD-18030.

## Data Availability

Supplementary Movies are available at: https://doi.org/10.6084/m9.figshare.23244299.v1.

## Supporting Information Available

Single-particle analysis of the BfrB data set on a Quantifoil grid with/without graphene, under conditions where the beam (900 nm) was smaller than the hole (1.2 μm) and not exposing the supporting foil/the beam (1.9 μm) was larger than the hole (1.2 μm), Rosenthal–Henderson *b* factor plots showing resolution versus number of particles of all four data sets, and captions for supplementary movies (PDF)
Supplementary movies as described in the text (ZIP)
