# Supplementary material for: Charging of Vitreous Samples in Cryogenic Electron Microscopy Mitigated by Graphene
Source: ACS Nano. 2023 Aug 2;17(16):15836–46. doi: 10.1021/acsnano.3c03722 (PMC10448747; doi:10.1021/acsnano.3c03722)
Supplement: Supplementary file 1 — nn3c03722_si_001.pdf [file nn3c03722_si_001.pdf]

Supporting information: Charging of vitreous samples in cryogenic electron microscopy mitigated by graphene

*Yue Zhang[1, ‡, *], J. Paul van Schayck[1, ‡], Adrián Pedrazo-Tardajos[2,3], Nathalie Claes[2,3], Willem E.M. Noteborn[4], Peng-Han Lu[5], Hans Duimel[1], Rafal E. Dunin-Borkowski[5, *], Sara Bals[2,3], Peter J. Peters[1], Raimond B.G. Ravelli[1, †]*

[1] Maastricht MultiModal Molecular Imaging Institute (M4i), Maastricht University, 6200 MD Maastricht, The Netherlands

[2] Electron Microscopy for Materials Science (EMAT), University of Antwerp, Antwerp 2020, Belgium

[3] NANOlab Center of Excellence, University of Antwerp, 2020 Antwerp, Belgium

[4] Supramolecular and Biomaterials Chemistry, Leiden Institute of Chemistry, Leiden University, 2300 RA Leiden, The Netherlands

[5] Ernst Ruska-Centre for Microscopy and Spectroscopy with Electrons and Peter Grünberg Institute, Forschungszentrum Jülich, 52425 Jülich, Germany

[†] In Memoriam: Raimond B.G. Ravelli, 25 March 1968 – 30 June 2023.

* Corresponding author: Yue Zhang: yue.zhang@maastrichtuniversity.nl

Rafal E. Dunin-Borkowski: r.dunin-borkowski@fz-juelich.de

‡ These two authors contributed equally.

Supplementary Figures

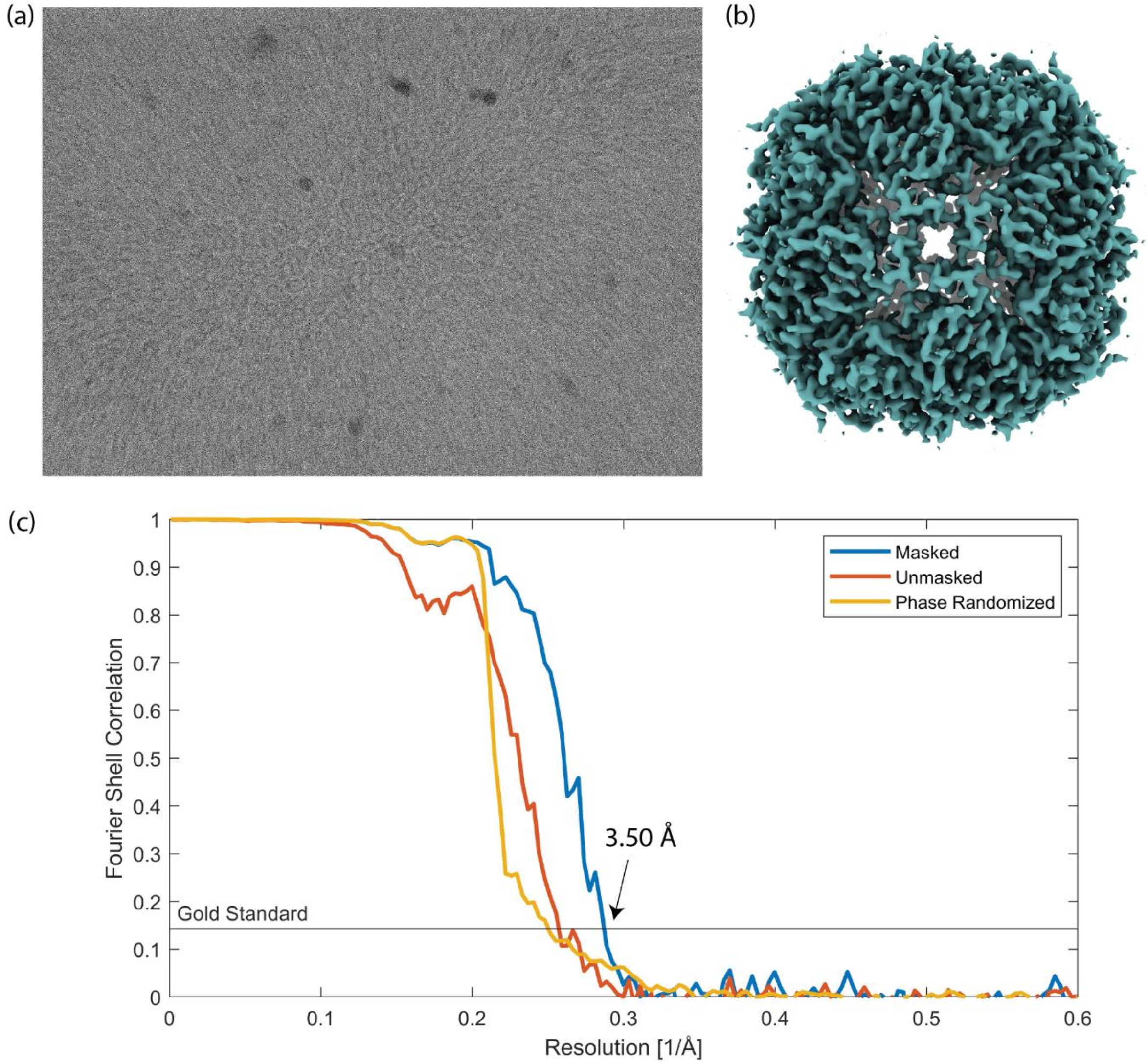

Supplementary Figure 1. Single-particle analysis of the BfrB data set on a Quantifoil grid without graphene, under conditions where the beam (900 nm) was smaller than the hole (1.2 μm) and not exposing the supporting foil. a) A micrograph of highly concentrated (50 mg/mL) BfrB in vitreous ice. b) 3D reconstruction from 85,707 particles at 3.50 Å resolution. c) Gold-standard Fourier shell correlation (FSC) before (red line) and after (blue line) masking, and the phase-randomised FSC (yellow line).

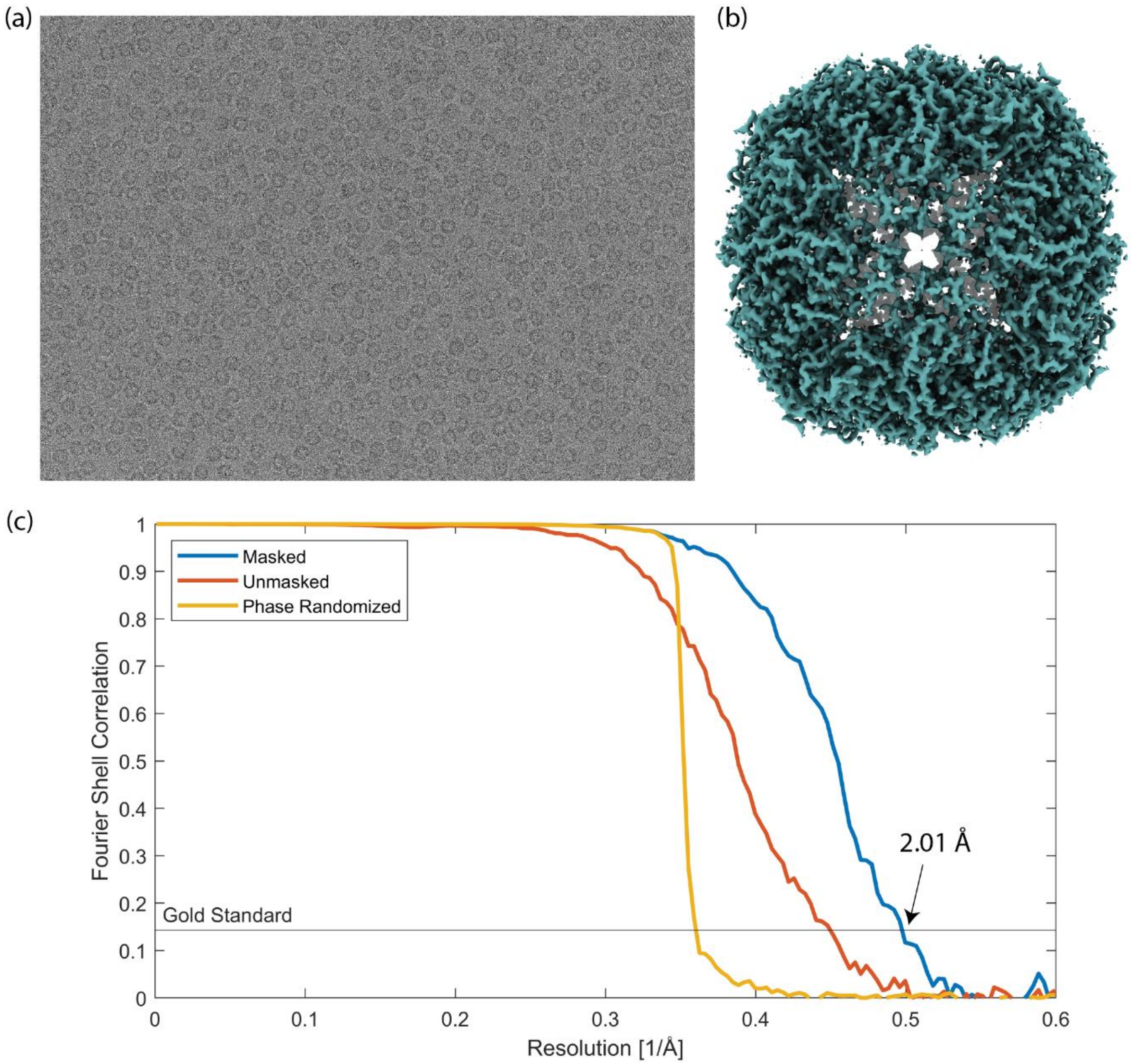

Supplementary Figure 2. Single-particle analysis of BfrB data set on Quantifoil grids with a graphene layer, under conditions where the beam (900 nm) was smaller than the hole (1.2 µm) and not exposing the supporting foil. a) A micrograph of diluted (5 mg/mL) BfrB in vitreous ice. b) 3D reconstruction from 146,626 particles at 2.01 Å resolution. c) Gold-standard Fourier shell correlation (FSC) before (red line) and after (blue line) masking, and the phase-randomised FSC (yellow line).

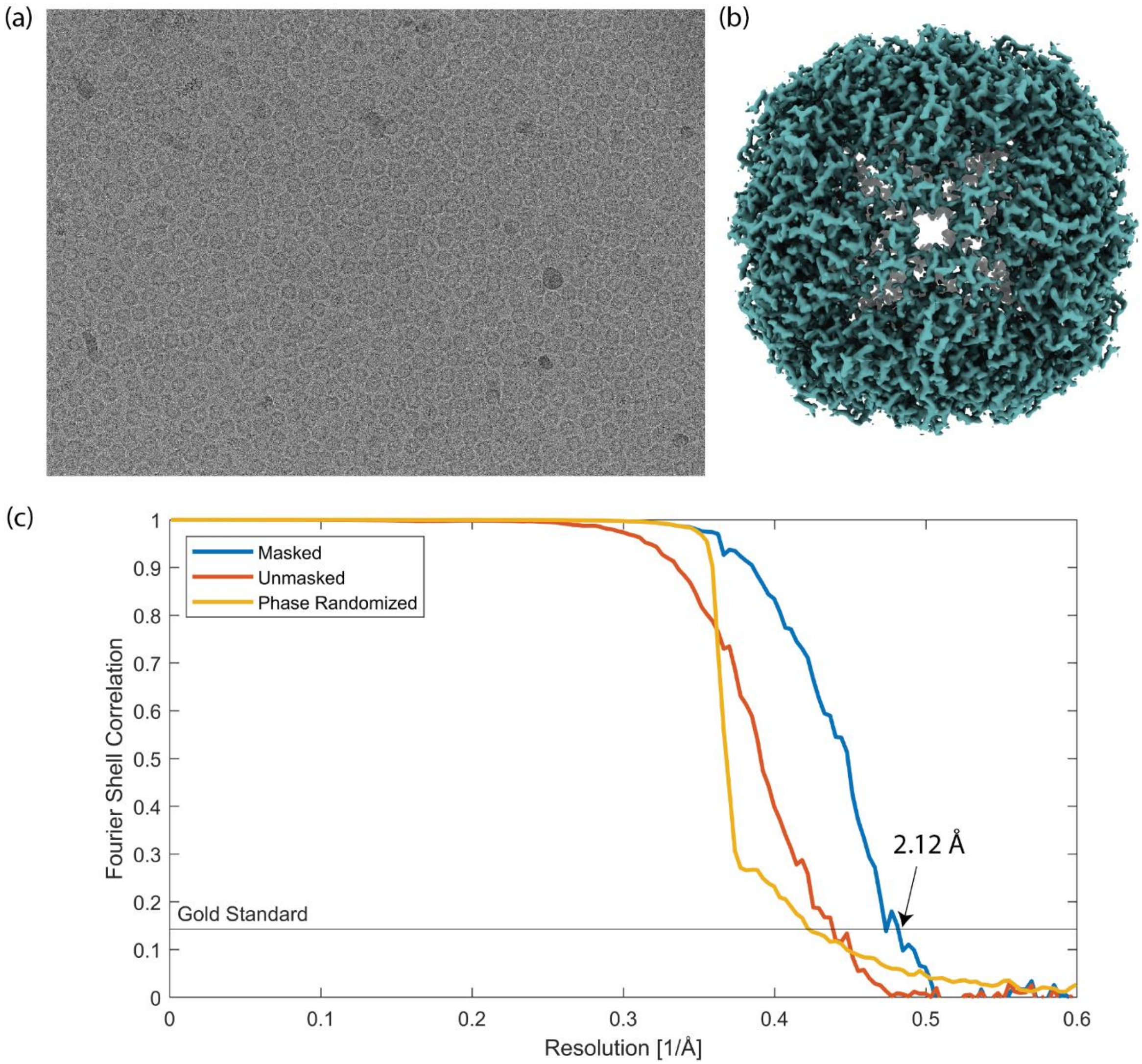

Supplementary Figure 3. Single-particle analysis of BfrB data set on normal Quantifoil grid without graphene, under conditions where the beam (1.9 μm) was larger than the hole (1.2 μm). a) A micrograph of highly concentrated (50 mg/mL) BfrB in vitreous ice. b) 3D reconstruction from 596,238 particles at 2.12 Å resolution. c) Gold-standard Fourier shell correlation (FSC) before (red line) and after (blue line) masking, and the phase-randomised FSC (yellow line).

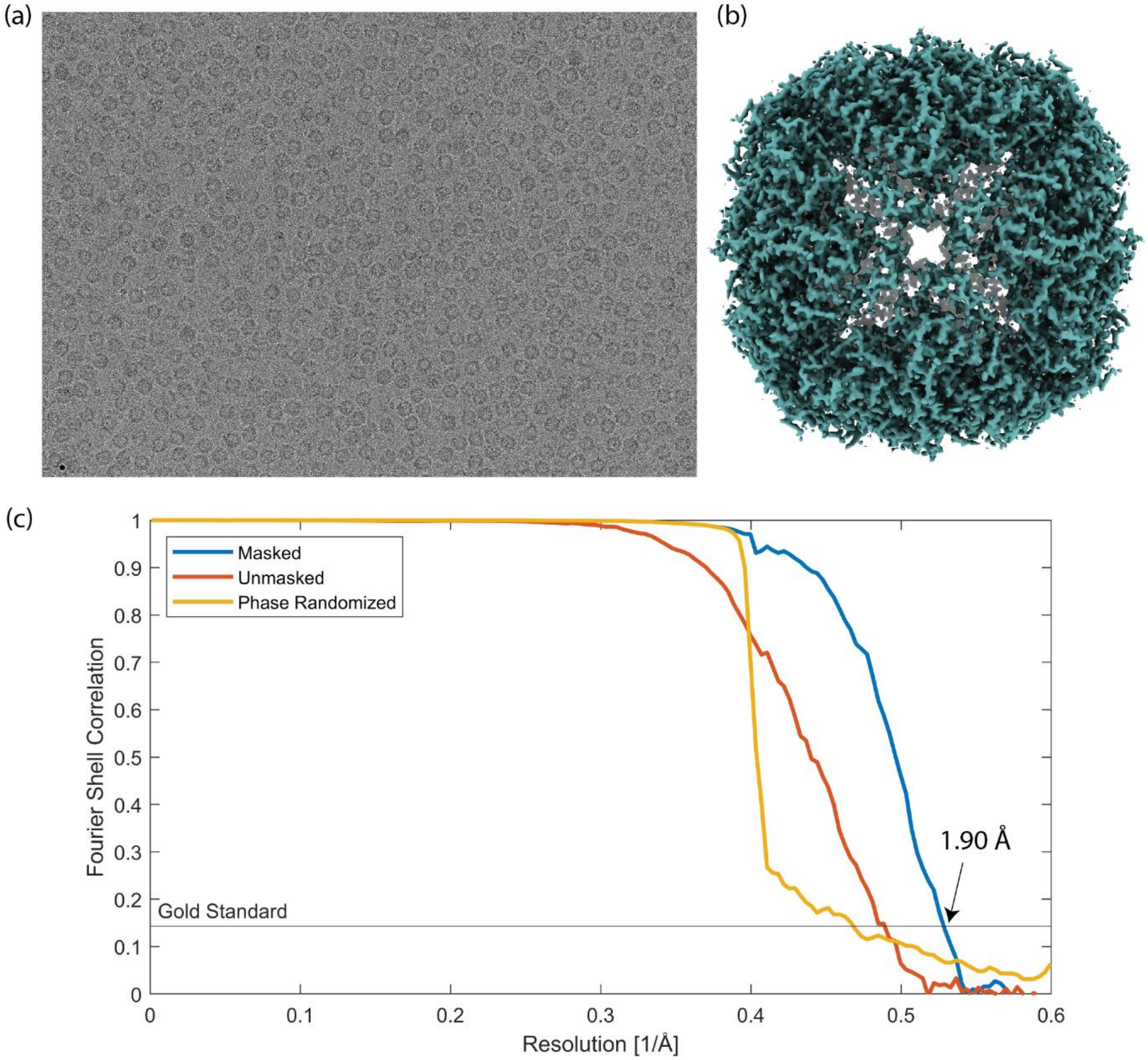

Supplementary Figure 4. Single-particle analysis of BfrB data set on Quantifoil grids with a graphene layer, under conditions where the beam (1.9 µm) was larger than the hole (1.2 µm). a) A micrograph of diluted (5 mg/mL) BfrB in vitreous ice. b) 3D reconstruction from 494,154 particles at 1.90 Å resolution. c) Gold-standard Fourier shell correlation (FSC) before (red line) and after (blue line) masking, and the phase-randomised FSC (yellow line).

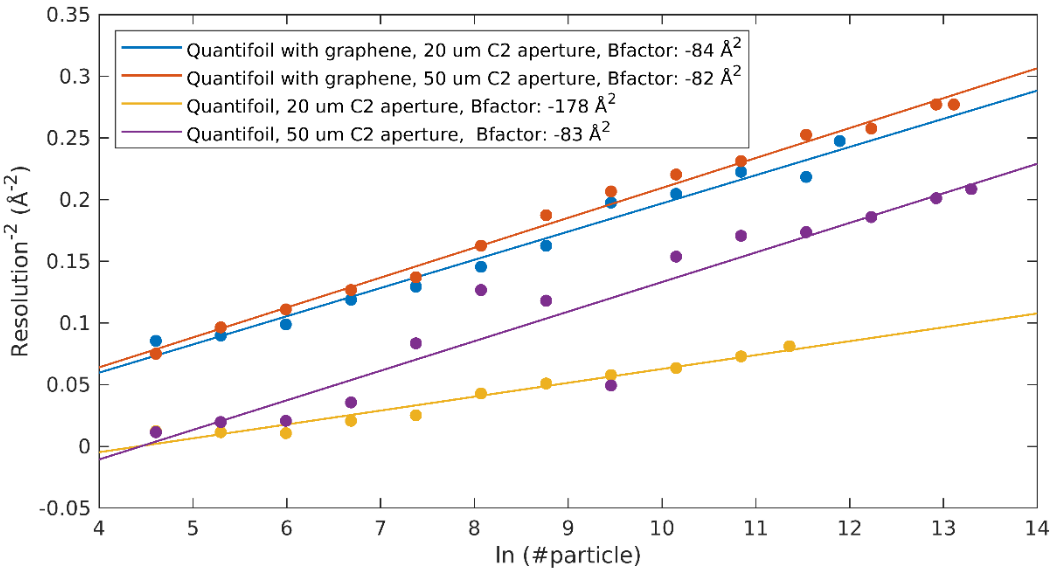

Supplementary Figure 5. The Rosenthal-Henderson B-factor plot showing resolution versus number of particles. B-factors were estimated by fitting a straight line through the inverse of the resolution squared versus the natural logarithm of the number of particles for a range random subsets of full particle lwast. (Rosenthal & Henderson, 2003).

Captions for supplementary movies

Supplementary Movies are available at:

https://www.dropbox.com/sh/sb8wval7cxt7v1p/AACONjlQgO8s_lLtJmKPYuEYa?dl=0

Supplementary Movie 1: Effect of specimen charging on Quantifoil in overfocus condition shown by DIFF images. The beam was parallel on vitreous specimen with the beam diameter (800 nm) smaller than the hole diameter (1.2 um). The electron beam flux on the sample was at 0.38 e^-/Å^2/s. The beam was blocked with the pre-specimen beam shutter before exposing the sample for recording showing as the black frames at the beginning of the movie.

Supplementary Movie 2: Effect of specimen charging on UltrAuFoil in overfocus condition shown by DIFF images. The beam was parallel on vitreous specimen with the beam diameter (800 nm) smaller than the hole diameter (1.2 um). The electron beam flux on the sample was at 0.38 e^-/Å^2/s. The beam was blocked with the pre-specimen beam shutter before exposing the sample for recording showing as the black frames at the beginning of the movie.

Supplementary Movie 3: Effect of specimen charging on Quantifoil in underfocus condition shown by DIFF images. The beam was parallel on vitreous specimen with the beam diameter (800 nm) smaller than the hole diameter (1.2 um). The electron beam flux on the sample was at 0.38 e^-/Å^2/s. The beam was blocked with the pre-specimen beam shutter before exposing the sample for recording showing as the black frames at the beginning of the movie.

Supplementary Movie 4: Effect of specimen charging on UltrAuFoil in underfocus condition shown by DIFF images. The beam was parallel on vitreous specimen with the beam diameter (800

nm) smaller than the hole diameter (1.2 um). The electron beam flux on the sample was at 0.38 $e^-/Å^2/s$. The beam was blocked with the pre-specimen beam shutter before exposing the sample for recording showing as the black frames at the beginning of the movie.

Supplementary Movie 5: Effect of specimen charging on cross-grating in overfocus condition shown by DIFF images. The beam was parallel with the beam diameter of 800 nm. The electron beam flux on the sample was at 0.38 $e^-/Å^2/s$. The beam was blocked with the pre-specimen beam shutter before exposing the sample for recording showing as the black frames at the beginning of the movie.

Supplementary Movie 6: Effect of specimen charging on cross-grating in underfocus condition shown by DIFF images. The beam was parallel with the beam diameter of 800 nm. The electron beam flux on the sample was at 0.38 $e^-/Å^2/s$. The beam was blocked with the pre-specimen beam shutter before exposing the sample for recording showing as the black frames at the beginning of the movie.

Supplementary Movie 7: Effect of specimen charging on Quantifoil in overfocus condition upon stage move shown by DIFF images. The beam was parallel on vitreous specimen with the beam diameter (800 nm) smaller than the hole diameter (1.2 um). The electron beam flux on the sample was at 0.38 $e^-/Å^2/s$. The beam was blocked with the pre-specimen beam shutter before exposing the sample for recording showing as the black frames at the beginning of the movie. The beam was completely within the foil hole on ice at the beginning of irradiation. After 5 s, the beam partially moved to one side of the carbon foil, followed by movement onto the carbon foil for 5 s. The beam then moved back to the edge of the foil for 5 s, then to the foil hole for another 5 s. The beam quickly scanned through the foil and remained at the edge of the carbon foil.

Supplementary Movie 8: Effect of specimen charging on Quantifoil in underfocus condition upon stage move shown by DIFF images. The beam was parallel on vitreous specimen with the beam diameter (800 nm) smaller than the hole diameter (1.2 um). The electron beam flux on the sample was at 0.38 $e^-/Å^2/s$. The beam was blocked with the pre-specimen beam shutter before exposing the sample for recording showing as the black frames at the beginning of the movie. The beam was completely within the foil hole on ice at the beginning of irradiation. After 5 s, the beam partially moved to one side of the carbon foil, followed by movement onto the carbon foil for 5 s. The beam then moved back to the edge of the foil for 5 s, then to the foil hole for another 5 s. The beam quickly scanned through the foil and remained at the edge of the carbon foil.

Supplementary Movie 9: Effect of specimen charging on UltrAuFoil in overfocus condition upon stage move shown by DIFF images. The beam was parallel on vitreous specimen with the beam diameter (800 nm) smaller than the hole diameter (1.2 um). The electron beam flux on the sample was at 0.38 $e^-/Å^2/s$. The beam was blocked with the pre-specimen beam shutter before exposing the sample for recording showing as the black frames at the beginning of the movie. The beam was completely within the foil hole on ice at the beginning of irradiation. After 5 s, the beam partially moved to one side of the gold foil, followed by movement onto the gold foil for 5 s. The beam then moved back to the edge of the foil for 5 s, then to the foil hole for another 5 s. The beam quickly scanned through the foil and remained at the edge of the gold foil.

Supplementary Movie 10: Effect of specimen charging on UltrAuFoil in underfocus condition upon stage move shown by DIFF images. The beam was parallel on vitreous specimen with the beam diameter (800 nm) smaller than the hole diameter (1.2 um). The electron beam flux on the sample was at 0.38 $e^-/Å^2/s$. The beam was blocked with the pre-specimen beam shutter before

exposing the sample for recording showing as the black frames at the beginning of the movie. The beam was completely within the foil hole on ice at the beginning of irradiation. After 5 s, the beam partially moved to one side of the gold foil, followed by movement onto the gold foil for 5 s. The beam then moved back to the edge of the foil for 5 s, then to the foil hole for another 5 s. The beam quickly scanned through the foil and remained at the edge of the gold foil.

Supplementary Movie 11: Effect of specimen charging on graphene-coated Quantifoil in overfocus condition upon stage move shown by DIFF images. The beam was parallel on vitreous specimen with the beam diameter (800 nm) smaller than the hole diameter (1.2 um). The electron beam flux on the sample was at 0.38 $e^{-}/Å^{2}/s$. The beam was blocked with the pre-specimen beam shutter before exposing the sample for recording showing as the black frames at the beginning of the movie.

Supplementary Movie 12: Effect of specimen charging on graphene-coated Quantifoil in underfocus condition upon stage move shown by DIFF images. The beam was parallel on vitreous specimen with the and a beam diameter (800 nm) smaller than the hole diameter (1.2 um). The electron beam flux on the sample was at 0.38 $e^{-}/Å^{2}/s$. The beam was blocked with the pre-specimen beam shutter before exposing the sample for recording showing as the black frames at the beginning of the movie.

Supplementary Movie 13: Effect of specimen charging on graphene-coated UltrAuFoil in overfocus condition upon stage move shown by DIFF images. The beam was parallel on vitreous specimen with the and a beam diameter (800 nm) smaller than the hole diameter (1.2 um). The electron beam flux on the sample was at 0.38 $e^{-}/Å^{2}/s$. The beam was blocked with the pre-specimen

beam shutter before exposing the sample for recording showing as the black frames at the beginning of the movie.

Supplementary Movie 14: Effect of specimen charging on graphene-coated UltrAuFoil in underfocus condition upon stage move shown by DIFF images. The beam was parallel on vitreous specimen with the and a beam diameter (800 nm) smaller than the hole diameter (1.2 um). The electron beam flux on the sample was at 0.38 $e^{-}/Å^{2}/s$. The beam was blocked with the pre-specimen beam shutter before exposing the sample for recording showing as the black frames at the beginning of the movie.

Supplementary Movie 15: Effect of specimen charging on graphene-coated Quantifoil in overfocus condition upon stage move shown by DIFF images. The beam was parallel on vitreous specimen with the beam diameter (800 nm) smaller than the hole diameter (1.2 um). The electron beam flux on the sample was at 0.38 $e^{-}/Å^{2}/s$. The beam was blocked with the pre-specimen beam shutter before exposing the sample for recording showing as the black frames at the beginning of the movie. The beam was completely within the foil hole on ice at the beginning of irradiation. The stage movement simulates the beam transitioning from the sample to the carbon foil.

Supplementary Movie 16: Effect of specimen charging on graphene-coated Quantifoil in underfocus condition upon stage move shown by DIFF images. The beam was parallel on vitreous specimen with the beam diameter (800 nm) smaller than the hole diameter (1.2 um). The electron beam flux on the sample was at 0.38 $e^{-}/Å^{2}/s$. The beam was blocked with the pre-specimen beam shutter before exposing the sample for recording showing as the black frames at the beginning of the movie. The beam was completely within the foil hole on ice at the beginning of irradiation. The stage movement simulates the beam transitioning from the sample to the carbon foil.

Supplementary Movie 17: Effect of specimen charging on graphene-coated UltrAuFoil in overfocus condition upon stage move shown by DIFF images. The beam was parallel on vitreous specimen with the and a beam diameter (800 nm) smaller than the hole diameter (1.2 um). The electron beam flux on the sample was at 0.38 $e^{-}/Å^{2}/s$. The beam was blocked with the pre-specimen beam shutter before exposing the sample for recording showing as the black frames at the beginning of the movie. The beam was completely within the foil hole on ice at the beginning of irradiation. The stage movement simulates the beam transitioning from the sample to the gold foil.

Supplementary Movie 18: Effect of specimen charging on graphene-coated UltrAuFoil in underfocus condition upon stage move shown by DIFF images. The beam was parallel on vitreous specimen with the and a beam diameter (800 nm) smaller than the hole diameter (1.2 um). The electron beam flux on the sample was at 0.38 $e^{-}/Å^{2}/s$. The beam was blocked with the pre-specimen beam shutter before exposing the sample for recording showing as the black frames at the beginning of the movie. The beam was completely within the foil hole on ice at the beginning of irradiation. The stage movement simulates the beam transitioning from the sample to the gold foil.

Supplementary Movie 19: Effect of specimen charging on Quantifoil in imaging mode. A micrograph of the BfrB sample with a concentration of 50 mg/ml on 1.2/1.3 Quantifoil grids was obtained at a magnification of 78,000× using a Falcon III at 200 kV. The data were collected with the beam not hitting the carbon foil, with a flux of 30 e-/Å2/s for 1 s using a 20 um aperture (beam diameter of 800 nm), and then repeated at the same position using a 50 um aperture (beam diameter of 1.9 um). A movie was generated by performing a running average (1-3, 2-4, …) of the fractions (40 fractions/s) and applying a Gaussian function (sigma = 3). No objective aperture was used.

Supplementary Movie 20: Effect of specimen charging on Quantifoil in imaging mode. A micrograph of the BfrB sample with a concentration of 50 mg/ml on 1.2/1.3 Quantifoil grids was obtained at a magnification of 78,000× using a Falcon III at 200 kV. The data was collected with the beam not hitting the carbon foil, with a flux of 30 e-/Å2/s for 1 s using a 50 um aperture (beam diameter of 1.9 um), and then repeated at the same position using a 20 um aperture (beam diameter of 800 nm). A movie was generated by performing a running average (1-3, 2-4, …) of the fractions (40 fractions/s) and applying a Gaussian function (sigma = 3). No objective aperture was used.

Supplementary Movie 21: Effect of specimen charging on graphene-coated Quantifoil in imaging mode. A micrograph of the BfrB sample with a concentration of 5 mg/ml on 1.2/1.3 Quantifoil grids was obtained at a magnification of 78,000× using a Falcon III at 200 kV. The data was collected with the beam not hitting the carbon foil, with a flux of 30 e-/Å2/s for 1 s using a 20 um aperture (beam diameter of 800 nm), and then repeated at the same position using a 50 um aperture (beam diameter of 1.9 um). A movie was generated by performing a running average (1-3, 2-4, …) of the fractions (40 fractions/s) and applying a Gaussian function (sigma = 3). No objective aperture was used.

Supplementary Movie 22: Effect of specimen charging on graphene-coated Quantifoil in imaging mode. A micrograph of the BfrB sample with a concentration of 5 mg/ml on 1.2/1.3 Quantifoil grids was obtained at a magnification of 78,000× using a Falcon III at 200 kV. The data was collected with the beam not hitting the carbon foil, with a flux of 30 e-/Å2/s for 1 s using a 50 um aperture (beam diameter of 1.9 um), and then repeated at the same position using a 20 um aperture (beam diameter of 800 nm). A movie was generated by performing a running average (1-

3, 2-4, …) of the fractions (40 fractions/s) and applying a Gaussian function (sigma = 3). No objective aperture was used.
